# The lipid-binding PX domain of RRC-1 (ARHGAP32/33) is required for optimal assembly and function of integrin adhesion complexes at the muscle cell boundary in *C. elegans*

**DOI:** 10.1093/g3journal/jkag143

**Published:** 2026-06-01

**Authors:** Sara Sagadiev, Isabel Martin, Nahum Arefeayne, Yuchu Wang, Robert Hudson, Olga Mayans, Hiroshi Qadota, Guy M Benian

**Affiliations:** Department of Pathology, Emory University, Atlanta, GA 30322, United States; Department of Biology, University of Konstanz, 78457 Konstanz, Germany; Department of Pathology, Emory University, Atlanta, GA 30322, United States; Department of Pathology, Emory University, Atlanta, GA 30322, United States; Department of Pathology, Emory University, Atlanta, GA 30322, United States; Department of Biology, University of Konstanz, 78457 Konstanz, Germany; Department of Pathology, Emory University, Atlanta, GA 30322, United States; Department of Pathology, Emory University, Atlanta, GA 30322, United States

**Keywords:** *C. elegans*, muscle, integrin adhesion complexes, PX domain, lipid binding, RhoGAP domain

## Abstract

Integrin adhesion complexes (IACs) are a network of many proteins that serve as anchors of the cell to the extracellular matrix (ECM). In muscle, IACs located at costameres also serve to transmit the force of muscle contraction to the outside of the cell. We have reported that IACs, which are found at the bases of dense bodies and M-lines, and at muscle cell boundaries (MCB) in *C*aenorhabditis *elegans* muscle, require the RacGEF PIX-1 for their proper assembly or maintenance. We have reported that a RacGAP for the PIX pathway is RRC-1, which is in a complex with PIX-1, and that RRC-1 is required for assembly or maintenance of IACs at MCBs. Our previous studies suggested that RRC-1 might be associated with the muscle cell membrane, and here we present evidence that this occurs via its PX domain, a domain that is known to bind to membrane phosphoinositides (PIPs). We predict the existence of a PX domain based on bioinformatic analysis and AlphaFold3, which includes conserved residues characteristic of most PX domains and a PIP-binding site. This region of RRC-1 binds to phosphoinositides in vitro. Analysis of a nematode strain that has an in-frame deletion of the PX domain generated by CRISPR/Cas9 indicates that normal localization of RRC-1 to the MCB requires both its PX domain and the PIX scaffold protein GIT-1. Lastly, overexpression experiments suggest that both the PX domain and the RhoGAP domain of RRC-1 each contribute to the proper function of RRC-1 at the MCB. Our study highlights the importance of RRC-1's lipid interactions at the cell membrane for the proper assembly and function of IACs in *C. elegans* muscle.

## Graphical Abstract

**Graphical Abstract:**
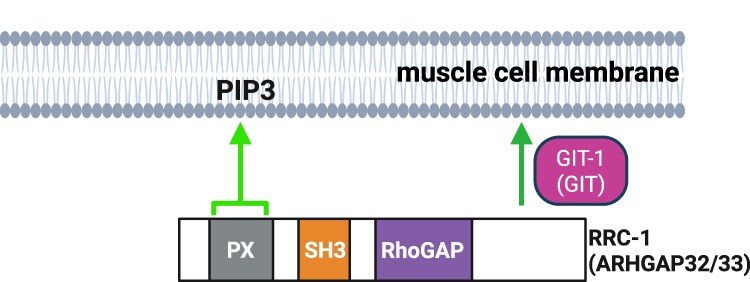

## Introduction

Integrin adhesion complexes (IACs), often referred to as focal adhesions, consist of the transmembrane α/β heterodimeric integrin and hundreds of other proteins, intracellular proteins associated with the cytoplasmic tail, and extracellular matrix (ECM) proteins associated with the extracellular portion of integrin (Anthis and Campbell 2011, Bachir et al. 2014). Dynamic assembly and disassembly of IACs are crucial for migrating cells during development, inflammation, and cancer, and for stationary cells to attach to the ECM (Horton et al. 2015). In striated muscle, which includes both skeletal and cardiac muscle, myofibrils at the periphery of the cell are attached to the cell membrane and ECM via “costameres,” muscle-specific IACs (Ervasti 2003). Costameres function to anchor the muscle cell to the ECM and transmit the force of muscle contraction to the outside of the cell. In the genetic model organism *Caenorhabditis elegans*, the major striated muscle is localized to the body wall, and through it, cyclical contraction and relaxation permit the whole nematode to move (Benian and Epstein 2011). The organization of its striated muscle is like other animals, where the thin filaments attach to Z-disk-like structures, called dense bodies, and thick filaments are cross-linked at M-lines. In *C. elegans* muscle, specific IACs are located at M-lines, dense bodies, and muscle cell boundaries (MCB) with the base consisting of integrins and a core set of proteins along with site-specific proteins (Gieseler et al. 2017; Qadota et al. 2017). Our laboratory was the first to report that the PIX signaling pathway has an important role in striated muscle (Moody et al. 2020). The *C. elegans* protein PIX-1 is required for the assembly or stability of IACs at the MCB. PIX is a RacGEF that activates the small GTPases Rac and Cdc42 by promoting the exchange of GTP for GDP (Mosaddeghzadeh and Ahmadian 2021). GTP-Rac binds to and activates effector protein kinases (called PAKs), which then phosphorylate unidentified substrates. In some still unknown way, activation of this pathway promotes the assembly of IACs. Loss-of-function mutations in any component of the known PIX pathway result in a similar phenotype—loss of assembly of IACs at the MCB. Moreover, either deficiency or overexpression of PIX-1 results in disrupted MCBs and decreased muscle function, indicating that the level of PIX-1 needs to be tightly controlled.

Rho family GTPases (Rho, Rac, Cdc42) cycle between active (GTP-bound) and inactive (GDP-bound) states. In the PIX pathway, activation occurs by PIX. Inactivation occurs via so-called GTPase-activating proteins (GAPs), which promote the hydrolysis of GTP to GDP. We have reported the identification of the first RacGAP for the PIX pathway in any organism or cell type, called RRC-1 (Moody et al. 2024). Loss of function of *rrc-1* results in reduced localization of IACs to the MCB, disorganization of M-lines and dense bodies, and reduced whole animal locomotion. Multiple lines of genetic and cell biologic evidence suggest that RRC-1 is part of the PIX-1 pathway: (i) each single mutant results in a similar defect at the MCB; (ii) PIX-1 and RRC-1 each localize to the MCB; (iii) localization of RRC-1 to the MCB depends on PIX-1 and vice versa; and (iv) RRC-1 exists in a complex with PIX-1.

Several pieces of data suggest that RRC-1 might be membrane-associated (Moody et al. 2024). First, in addition to localizing to the MCB, RRC-1 is also localized at what seems to be the bases of the M-lines and dense bodies, but in a fuzzy and indistinct manner reminiscent of the localization of the ECM protein UNC-52 (perlecan). Second, to demonstrate co-IP of RRC-1 with PIX-1, it was necessary to use a small amount of the ionic detergent, deoxycholate, in addition to a nonionic detergent to extract RRC-1 from worms. Third, the human orthologs for RRC-1, ARHGAP32, and ARHGAP33 have predicted phox homology (PX) domains. PX domains are known to bind to phosphoinositides (PIPs) and target proteins to membranes (Chandra and Collins 2019). However, in our previous report on RRC-1 (Moody et al. 2024), we could not predict a PX domain in RRC-1 using PFAM and ProSite programs. Here, we report that we can now predict a PX domain by sequence, and by homology modeling, and that indeed this PX domain binds to PIPs. By generating an in-frame deletion of the PX domain, we find that it is required for normal worm locomotion. Moreover, optimal localization of RRC-1 to the MCB requires both its PX domain and the PIX-1 scaffold protein GIT-1. Finally, overexpression experiments indicate that both the PX domain and the RhoGAP domain each contribute to the function of RRC-1 at the MCB.

## Materials and methods

### Prediction of the existence of a PX domain in RRC-1 and generation and analysis of its 3D model

The 3D fold of the PX domain from RRC-1 (residues 41 to 146; UniProtKB Q20498-1) was modeled using AlphaFold3 (Abramson et al. 2024). The predicted model was compared to all protein entries at the RCSB Protein Data Bank (https://www.rcsb.org/) using the DALI server (http://ekhidna2.biocenter.helsinki.fi/dali/; Holm 2022). The top 10 experimental structures identified in this way were structurally aligned to the PX-RRC-1 domain using UCSF Chimera version 1.19 (https://www.cgl.ucsf.edu/chimera/; Pettersen et al. 2004). Matchmaker was used in its default settings (chain pairing: bb, Needleman-Wunsch using the BLOSUM-62, ss fraction: 0.3, gap open (HH/SS/other) 18/18/6, extend 1, ss matrix: (O, S): -6 (H, O): -6 (H, H): 6 (S, S): 6 (H, S): -9 (O, O): 4, iteration cutoff: 2) (Meng et al. 2006). A structure-based multiple sequence alignment was then generated from the superimposed models using UCSF Chimera's Match->Align function in default settings and alignment conservation scores were calculated using the built-in conservation annotation function, based on sequence identity and similarity of physico-chemical properties (Pettersen et al. 2004; Meng et al. 2006). The percentage of sequence identity in the Match->Align function was calculated with the reference sequence set to the RRC-1 PX domain.

### Expression and purification of GST, GST-RRC-1 (1 to 150), and 6xHis-RRC-1 (36 to 150)

To create a plasmid for bacterial expression of GST-RRC-1 (1 to 150 aa), the coding sequence for residues 1 to 150 was amplified by PCR using as template a full-length *rrc-1* cDNA (Moody et al. 2024), and the following primers:

Rrc-1 anti-N1: BamHI-ATG-

GCGGGATCCATGGAAGGCATCGAGGAATCA

Rrc-1 anti-N2: -150aa-XhoI

CGCCTCGAGTTAGCCACGAGAGTCAATTTCTAA

The amplified fragment was cloned into the BamHI and XhoI sites of pGEX-KK-1 and a clone was identified to be error-free by Sanger sequencing. To create a plasmid for bacterial expression of 6xHis-RRC-1 (36 to 150 aa), the coding sequence for residues 36 to 150 was amplified by PCR using as template a full-length *rrc-1* cDNA (Moody et al. 2024), and the following primers:

His-RRC-1-PX-1: NcoI-36aa-

GCGCCATGGCGTTCCATTATTCGTCTGTTGAATTG

His-RRC-1-PX-2: -150aa-STOP-XhoI

CGCCTCGAGTTAGCCACGAGAGTCAATTTCTAAAAATTTC

The amplified fragment was cloned into the NcoI and XhoI sites of pETM11 and a clone was identified to be error-free by Sanger sequencing.

pGEX-KK1, pGEX-KK1-RRC-1 (1 to 150), and pETM11-RRC-1 (36 to 150) were transformed into *E. coli* Rosetta 2 (DE3) and used to produce proteins using procedures described in Matsunaga et al. (2024) for the GST proteins, and in Qadota et al. (2018) for the 6His protein, from 1 liter of culture each. A Bradford Assay (Bio-Rad, Inc.) was used to determine the protein concentrations, and the proteins were stored on ice until used.

### Lipid protein overlay assay with a PIP strip

We used PIP Strip Membranes (Molecular Probes/Invitrogen), with a protocol recommended by the manufacturer. The strip was blocked with 25 mL of TBS-T+3% fatty acid-free BSA (EMD Millipore Corporation) for 1 h and was then incubated in 5 mL TBS-T+3% fatty acid-free BSA containing either GST-RRC-1 (1 to 150), or GST at a concentration of 0.5 µg/mL overnight at 4 °C. The membrane was then washed 3 times for 10 min each time with 20 mL of the TBS-T+3% BSA. We then incubated the strip with anti-GST (mouse monoclonal 26H1 from Cell Signaling Technology) at 1:1,000 dilution in TBS-T+3%BSA for 1 h at room temperature. This was followed by 3, 10-min washes, and then incubation with anti-mouse-HRP (GE Healthcare) at 1:5,000 dilution, and then finally 3, 10-min washes all with TBS-T+3% BSA. The reactions were detected by ECL and exposure to film.

### Mass spectrometry to determine lipids bound to 6xHis-RRC-1 (36 to 150)

Phospholipid molecular species were measured by LC-MS/MS at the Emory Integrated Metabolomics and Lipidomics Core (EIMLC; Emory University, Atlanta, GA, USA). Standard Lipidomix Splash Mix was purchased from Avanti Polar Lipids (Alabaster, AL, USA) and used as an internal standard, including 15:0–18:1d7 phosphatidic acid, 15:0–18:1d7 phosphatidylethanolamine, and 15:0–18:1d7 phosphatidylglycerol. 6xHis-RRC-1 (36 to 150 aa) was prepared as described above from two 500 mL cultures, giving a yield of ∼0.33 mg/mL and a total of ∼2.0 mg, which was dialyzed against phosphate buffer saline plus 0.7 mM TCEP, and provided to the EIMLC. The protein was extracted using a modified Bligh and Dyer lipid extraction (2:1 v/v methanol:chloroform). The organic phase was then collected and dried under nitrogen gas. Recovered lipids were reconstituted in 1 mL of 1:1 v/v chloroform:methanol, and 10 μL internal standard was spiked into the extract directly prior to injection into the mass spectrometer. Targeted lipidomic experiments were performed using a Sciex QTrap5500 using an enhanced MS1 scan (EMS) LC/MS method, whereby all peaks with a signal-to-noise ratio above 10 are fragmented for characterization (Lee et al. 2011). For chromatography, 20 μL of extracts were injected onto an Agilent C18 column, and lipids were resolved on a 10 min linear gradient whereby 60:40 acetonitrile (ACN): water was used as Solvent A and 90:10 2-Propanol (IPA): ACN was Solvent B. Both solvents contained 10 mM ammonium formate and 0.1% formic acid. Targeted lipids were quantified in μg/mg by dividing the peak area of the sample by the internal standard's response factor, calculated as the area signal/concentration signal.

### Liposome pelleting assay

Reconstituted lipid stocks (Avanti Polar Lipids) were combined and dried under argon to remove chloroform, followed by incubation in a vacuum desiccator for 1 h. Lipids were resuspended in buffer (20 mM HEPES, 100 mM NaCl, pH 7.5) to form multilamellar vesicles (MLVs) and incubated at 37 °C for 2 h. Purified 6xHis-RRC-1 (36 to 150 aa) protein was filtered (0.22 μm) and incubated with MLVs (3 μg protein, 15 μg lipids) at 4 °C for 1 h. Samples were centrifuged at 16,000 × *g* for 20 min at 4 °C to separate supernatant and pellet fractions. Pellets were resuspended in the same buffer, and all samples were mixed with Laemmli buffer containing 1% β-mercaptoethanol, boiled at 95 °C for 5 min, and resolved on 8% to 16% SDS-PAGE gels (Bio-Rad). Gels were stained with Coomassie Brilliant Blue R250, destained, and imaged.

### *C. elegans* strains

C. *elegans* were grown on NGM plates utilizing standard methods and maintained at 20 ^°^C (Brenner 1974). The following strains were used:

Wild type, Bristol (N2)

PHX4499, *rrc-1(syb4499)*, by CRISPR, which expresses RRC-1 with a C-terminal HA tag (described in Moody et al. 2024)

PHX10067, *rrc-1(syb4499  syb10067)*, by CRISPR, described below, which expresses RRC-1 with a C-terminal HA tag and an in-frame deletion of the PX domain (residues 36 to 143)

GB390, *rrc-1(syb4499)* after outcrossing 4X to wild type

GB391, *rrc-1(syb4499  syb10067)* after outcrossing 4X to wild type

GB377: *sfEx77* [myo-3p::RRC-1::HA; sur-5::GFP], overexpression in wild-type background of full-length RRC-1-HA in muscle from an extrachromosomal array. This strain was created by crossing in the extrachromosomal array [myo-3p::RRC-1::HA; sur-5::GFP], from strain GB372 (Moody et al. 2024), which had been used to rescue *rrc-1(ok1747*), into wild type.

GB378: *sfEx79* [myo-3p::ΔNterm-RRC-1::HA; sur-5::GFP], overexpression in wild-type background of in-frame deleted PX domain-RRC-1-HA from an extrachromosomal array. Generation of this transgenic strain is described below.

GB413: *sfEx78* [myo-3p::RRC-1 [R315M]-HA; sur-5::GFP], overexpression in wild-type background in muscle of full-length RRC-1-HA with the R315M mutation that inactivates the RhoGAP domain, and derived by crossing into wild type this extrachromosomal array from strain GB373 (Moody et al. 2024) that was used to attempt rescue of *rrc-1(ok1747*).

GB379: *sfIs28* [myo-3p::RRC-1::HA; sur-5::GFP], integrated array (from GB377) overexpressing in wild-type background full-length RRC-1-HA in muscle. The integration procedure is described below.

GB380: *sfIs29* [myo-3p::ΔNterm-RRC-1::HA; sur-5::GFP], integrated array (from GB378) overexpressing in wild-type background PX domain-deleted RRC-1-HA. The integration procedure is described below.

GB399: GB379 (*sfIs28* [myo-3p::RRC-1::HA; sur-5::GFP]), after outcrossing 3X to wild type

GB400: GB380 (*sfIs29* [myo-3p::ΔNterm-RRC-1::HA; sur-5::GFP]), after outcrossing 3X to wild type

### Generation of GB378 (transgenic overexpression of PX-deleted RRC-1)

To create pPD95.86-ΔPX-RRC-1-HA (deleted 1 to 140 aa), a cDNA fragment encoding residues 141 to 759 was amplified using a full-length rrc-1 cDNA (Moody et al. 2024) and the following primers:

rrc-1-10: SmaI-NheI-rrc-1a ATG-141-

GCGCCCGGGGCTAGCATGCTGAAATTTTTAGAAATTGAC

rrc-1-3: --(EcoRI)-HindIII

CGCAAGCTTCTCTGAATATTCGATTGAATTC

The amplified fragment was cloned into the SmaI and HindIII sites of pBluescript KS+ and a clone was identified by Sanger sequencing to be error-free was called pBS-RRC-1-103. EcoRI, HindIII fragment of pBS-RRC-1-49HA (Moody et al 2024) was cloned into pBS-RRC-1-103 EcoRI, HindIII sites, resulting pBS-ΔPX-RRC-1-HA. NheI fragment of pBS-ΔPX-RRC-1-HA was cloned into pPD95.86 NheI site, resulting pPD95.86-ΔPX-RRC-1-HA. To create GB378, pPD95.86-ΔPX-RRC-1-HA together with pTG96 (sur-5::gfp transformation marker) at a ratio of 1:10 was injected into wild type N2 strain, and screened for the presence of sur-5-GFP expression.

### Integration of transgenic arrays

Extrachromosomal arrays in strains GB377 and GB378 were integrated randomly into the genome by ultraviolet (UV) irradiation following the procedure of Mitani (1995) with some modifications (Peter Barret and Jenna Hill, personal communication). Briefly, 40 GFP-positive transgenic L4 hermaphrodites were picked onto 8 OP50 freshly seeded 6 cm plates, 5 worms onto each plate. The plates were then mutagenized in a UV crosslinker at a dose of 250 mJ/cm^2^ for 30 s of exposure with the lid of the plates off. Plates were then sealed with parafilm and stored at 20 °C until starvation estimated to be in 2 wk. Following starvation, the plates were chunked to new plates and numbered to track each integrant. A total of 11 chunked plates were made for each integrant (full-length RRC-1 and ΔNterminus of RRC-1). These plates were then stored in 20 °C and allowed to grow for 2 d. From these plates, a total of 100, L4 or earlier GFP+ transgenic worms were picked onto separate plates. After allowing to grow for 1 generation, plates were identified that had 100% of the progeny being GFP+, and this transmission rate continued through the F2 generation. One such plate was identified for each extrachromosomal array.

### Generation of PHX10067

The CRISPR/Cas9 procedures were carried out by SunyBiotech (www.sunybiotech.com). Details about the sgRNAs and repair template used are given in Supplementary Fig. 1.

### Worm locomotion assays

Swimming and crawling assays were performed on day 2 adults as described previously (Moody et al. 2024).

### RNAi knockdown of *git-1*

RNAi knockdown of *git-1* in GB390 and GB391 was performed as described previously (Moody et al. 2024).

### Western blots

The method of Hannak et al. (2002) was used to prepare total protein lysates from the CRISPR lines, *rrc-1(syb4499)* [RRC-1-HA], [ΔPX RRC-HA], and the transgenic lines that overexpress in a wild-type background, RRC-1-HA and ΔPX RRC-1-HA. Equal amounts of total protein were separated on 10% polyacrylamide-SDS- Laemmli gels, transferred to nitrocellulose membranes, reacted with anti-HA (rabbit monoclonal) at 1:1,000 dilution and then reacted with goat anti-rabbit immunoglobulin G conjugated to HRP (GE Healthcare) at 1:10,000 dilution, and visualized by ECL reagents.

### Fixation, immunostaining, and confocal microscopy of body wall muscle

Adult worms were fixed and immunostained using previously described methods (Nonet et al. 1993; Wilson et al. 2012). Primary antibodies were anti-HA (rabbit monoclonal C29F4 from Cell Signaling Technology) used at a 1:200 dilution, and the following antibodies used at 1:100 dilution: anti-PAT-6 (rat polyclonal; Warner et al. 2013); anti-UNC-95 (rabbit polyclonal Benian-13; Qadota et al. 2007), anti-MHC A (mouse monoclonal 5-6; Miller et al., 1983; purchased from University of Iowa Hybridoma Bank), anti-UNC-89 (rabbit polyclonal EU30; Benian et al. 1996), and anti-ATN-1 (mouse monoclonal MH35; Francis and Waterston 1991). Secondary antibodies were used at a 1:200 dilution, including anti-Rabbit Alexa 488, anti-Rat Alexa 594, and anti-Mouse Alexa 594 purchased from Invitrogen. Images were captured at room temperature with either (i) a Zeiss confocal system (LSM510) equipped with an Axiovert 100 M microscope with an Apochromat x63/1.4 numerical aperture oil immersion objective, or (ii) a Leica SP8 multiphoton upright microscope with a HC PL APO x63/1.4 numerical aperture oil immersion objective. The color balances were adjusted by using Adobe Photoshop (Adobe, San Jose, CA).

## Results

**Fig. 1.**
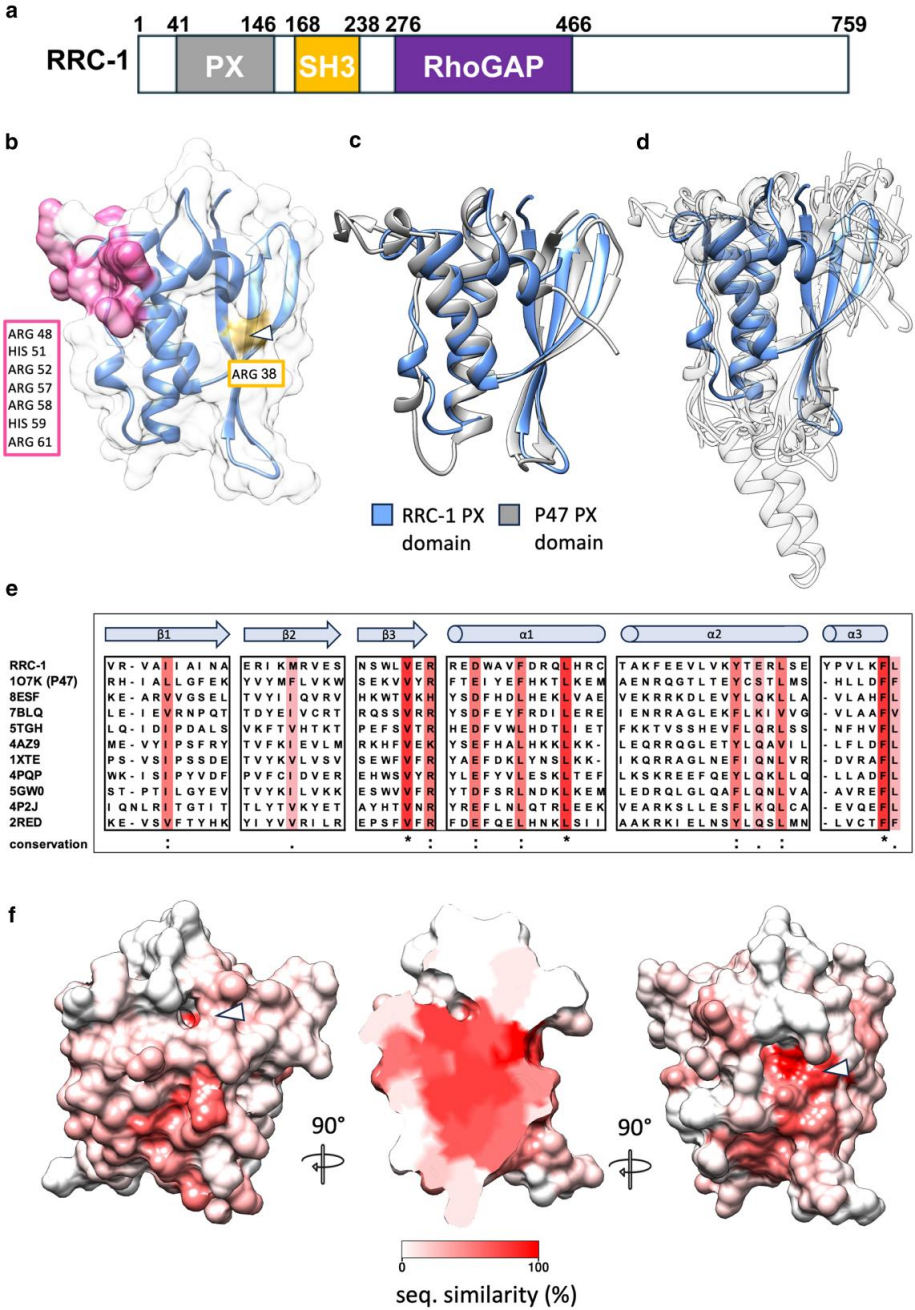
RRC-1 is predicted to comprise a canonical PX domain. a) Schematic representation of domains in RRC-1 predicted by NCBI Conserved Domains; b) predicted 3D-model of the RRC-1 PX-domain displayed both as ribbon representation and molecular surface. The surface representation is colored to show the cluster of basic amino acids at the secondary PIP2/PIP3 binding site (pink) and residue ARG38, part of the canonical PtdIns3P-binding site (yellow); c) the 3D-model of PX-RRC-1 (blue) is superimposed onto the experimental structure of the PX domain from P47phox (gray), the closest structural homolog. d) Superimposition of PX-RRC-1 (blue) and the top 10 closest PX domains identified using DALI (Holm 2022) (transparent ribbons). Despite low levels of sequence similarity, the main fold topology is strongly conserved with main differences being only present in loop regions. e) Structure-based sequence alignment of the secondary structure elements of the PX folds. For each sequence, PDB codes are provided. Note that the P47 PX domain is “107K (P47).” Conserved residues are colored in a white-to-red gradient according to increasing conservation levels (the complete structure-based sequence alignment is provided as Supplementary Fig. 3). f) RRC-1 PX domain colored by conservation as in (e). Conserved residues cluster on a deep cavity of the fold (indicated with a white arrowhead).

PX domains share little sequence similarity, so that the detection of such domains in proteins from sequence data alone can be difficult. Accordingly, to query the possible existence of a PX domain in RRC-1, a sequence-based search of the RCSB Protein Data Bank (https://www.rcsb.org) was performed as well as 3 different protein domain prediction programs: ProSite (https://prosite.expasy.org), PFAM (http://pfam.xfam.org), and NCBI Conserved Domains (https://www.ncbi.nlm.nih.gov/Structure/cdd/wrpsb.cgi). Of these approaches, only NCBI Conserved Domains predicted a PX domain in RRC-1, located at residues 41 to 146 (Fig. 1a). To validate that the identified sequence segment was compatible with a PX domain, we calculated its predicted 3D fold using AlphaFold3 (https://alphafoldserver.com; Abramson et al. 2024). The predicted model exhibited a high plDDT score (>90) in all regions other than loops, which indicates high prediction confidence (Supplementary Fig. 2). The model displayed a fold consisting of 3 antiparallel β-strands (β1 to β3) followed by 3 α-helices (α1 to α3) (Fig. 1b), as is typical of canonical PX-domains (Chandra and Collins 2019). This showed that the identified sequence segment in RRC-1 is compatible with the structurally conserved 3D fold of PX domains. Next, to determine whether the RRC-1 PX domain possesses functional features characteristic of PX domains, we assessed the presence of the canonical PtdIns3P-binding site, the polyproline motif ΦPxxPxK (Φ: hydrophobic residue), and the secondary PIP2/PIP3-binding site, as described for PX domains (Chandra et al. 2019). The canonical PtdIns3P-binding site was largely absent, with a single amino acid—R38—remaining conserved (Fig. 1b, yellow; Supplementary Fig. 3), and no polyproline sequence motif could be detected. However, the secondary PIP2/PIP3 binding site, which typically consists of a surface patch of basic amino acids vicinal to the primary PtdIns3P site, can be predictably matched to the presence of residues R48, H51, R52, R57, R58, H59, and R61 in that region of the fold (Fig. 1b, pink: Supplementary Fig. 3). This hints at the possibility that PX-RRC1 might have broad specificity for phospholipids and binds a range of these ligands.

To further confirm its PX-fold identity, we aimed to identify additional sequence determinants of the PX fold in the domain from RRC-1. For this, and since sequence-based approaches did not prove successful in identifying homology, we compared the calculated 3D-model to experimental structures of PX-domains available at the PDB using DALI as a search tool (http://ekhidna2.biocenter.helsinki.fi/dali; Holm 2022) and utilized the identified structural homologues to reveal conserved sequence loci. The closest structural homologue of the RRC-1 PX domain identified in this way was the PX domain from P47phox that displayed only minor structural differences (RMSD = 1.8 Å over 91 atom pairs) (Fig. 1c). To identify sequence conservation, the top 10 hits of the DALI search (*Z*-score >8; RMSD <3 Å) corresponding to the homologues most closely related to PX-RRC-1 were selected for comparison (see Supplementary Table 1). The 3D model of PX-RRC-1 and the 10 experimental structures so selected were superimposed, revealing a conserved fold with deviations only in loop regions (Fig. 1d). A structure-based sequence alignment of the superimposed structures that used UCSF Chimera (https://www.cgl.ucsf.edu/chimera; Pettersen et al. 2004) revealed low sequence identity levels (11 to 21%) for any given pair (Supplementary Fig. 3; Supplementary Table 1). Yet, several hydrophobic residues could be observed to be highly conserved (Fig. 1e). These residues have been reported to be characteristic of most PX domains (Seet and Hong 2006). Subsequently, we mapped this conservation onto the 3D model of the PX-RRC-1 domain and found that the conserved amino acids correspond largely to the protein hydrophobic core as well as forming a small cluster on the protein surface, localizing at a topographical deep cavity that penetrates the fold (Fig. 1f). Notably, residue R38 (Fig. 1b), part of the canonical PtdIns3P binding site, is located in this conserved surface patch, at the entry to the cavity. Whether this locus still acts as a phosphoinositide-binding site despite lacking complete PtdIns3P binding features is yet to be revealed. Deep cavities comparable to those modeled in PX-RRC-1 are observed in experimental 3D structures of other PX-domains, eg in sorting nexin 3 (PDB entry 7BLQ) and sorting nexin 14 (PDB 4PQP). So far, no function has been assigned to such cavities and their potential contribution to phosphoinositide binding is unknown. Taken together, our bioinformatics analyses lead us to conclude that RRC-1 contains a PX domain of broad phosphoinositide binding specificity.

**Fig. 2.**
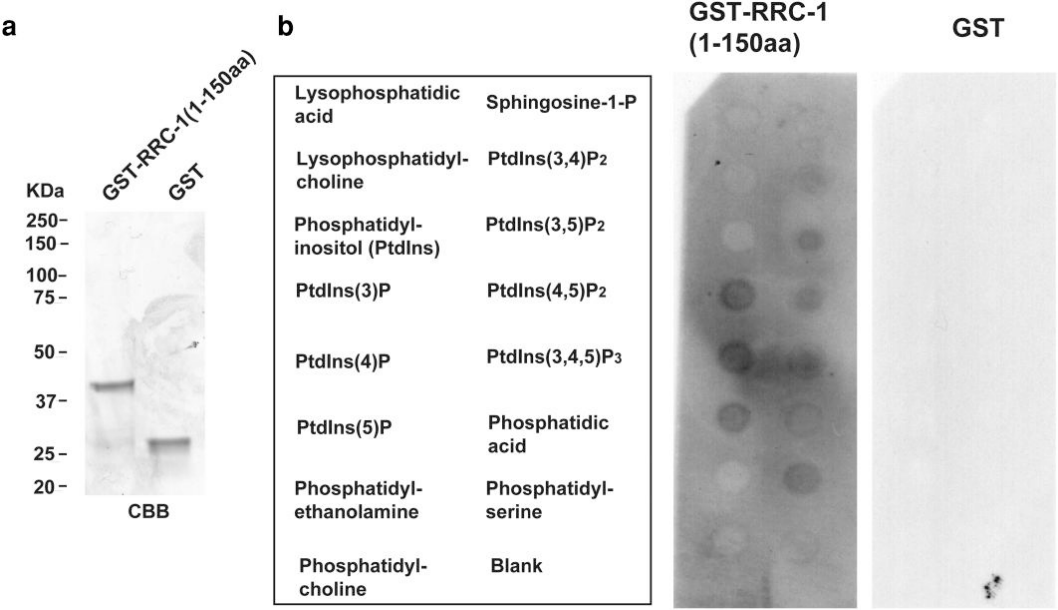
Protein-lipid overlay assay demonstrates that RRC-1 (1 to 150) binds to phosphoinositides. a) Coomassie Brilliant Blue (CBB) stained SDS-PAGE showing 2 µg of bacterially expressed GST-RRC-1 (1 to 150), and GST itself. Positions of molecular weight size markers are indicated. b) Results of incubating GST-RRC-1 (1 to 150) and GST with a PIP Thin Strip (ThermoFisher), and detection using antibodies to GST. Identities of spotted lipids are shown to the left.

To experimentally confirm the ability of the RRC-1 PX domain to bind to phosphoinositides, we used a blot (“PIP strip”) containing spots of various phosphoinositides to determine if the PX domain of RRC-1 can bind to phosphoinositides, and if so, which ones. We purified a bacterially expressed GST fusion protein that contains residues 1 to 150 of RRC-1 (Fig. 2a) to test for lipid binding. Figure 2b displays the results of incubating the strip with GST-RRC-1 (1 to 150), followed by detection with anti-GST antibodies. There is some binding to the following: phosphatidylinositol 3,5-diphosphate, phosphatidylinositol 3-phosphate, phosphatidylinositol 4,5-diphosphate, phosphatidyl 4-phosphate, phosphatidylinositol 3,4,5-triphosphate, phosphatidylinositol 5-phosphate, and phosphatidyl-serine. This was compared to a control PIP strip, which was incubated with GST protein with no resultant binding to any of the phosphoinositides present (Fig. 2b). The same general pattern of binding was found from 2 additional experiments using 2 separate PIP strips and an independently produced GST-RRC-1 (1 to 150) protein; binding to GST itself was never observed. One example of one of these additional experiments is shown in Supplementary Fig. 4. We conclude that the predicted PX domain of RRC-1 does have affinity for membrane-localized phosphoinositides.

**Fig. 3.**
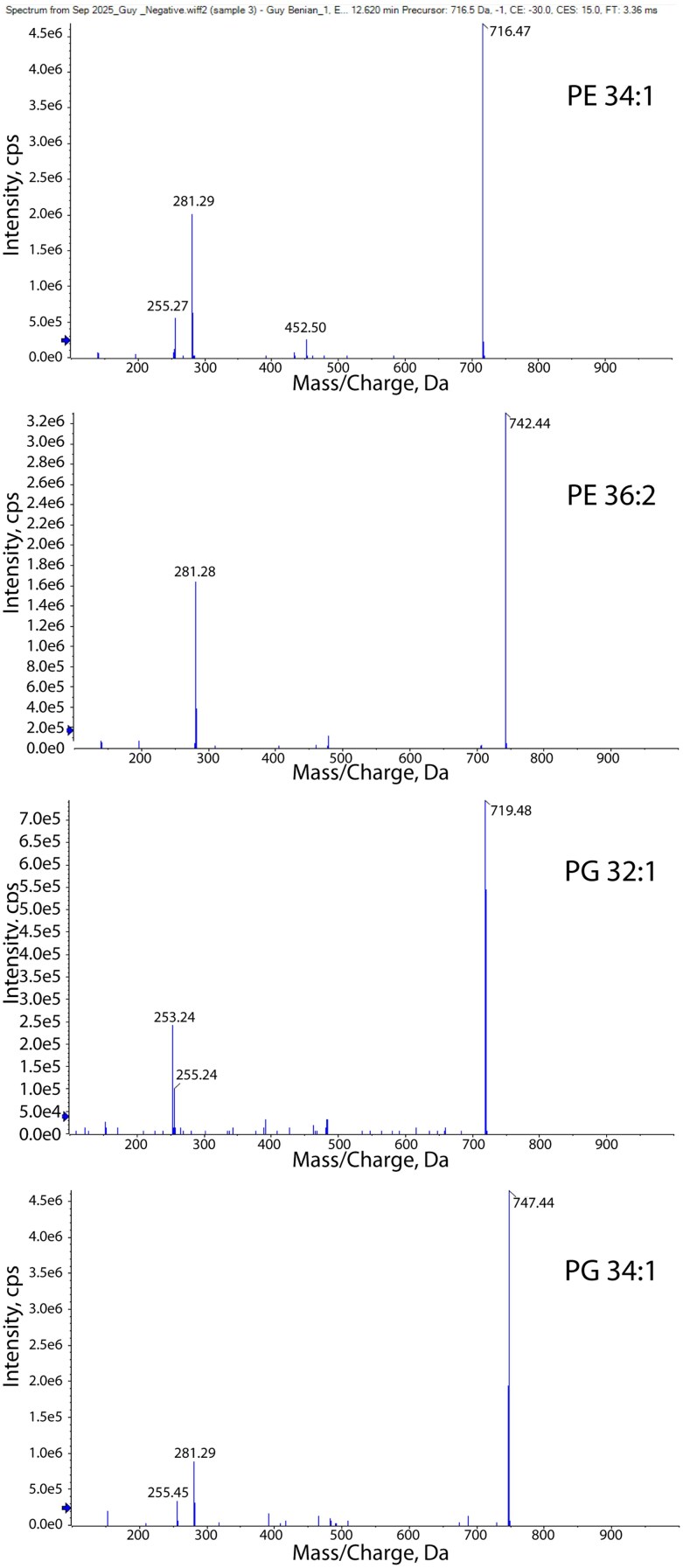
Phospholipids identified as bound to 6His-RRC-1 (36 to 150 aa) by LC–MS/MS. Phospholipids associated with recombinant 6His-RRC-1 (36 to 150 aa) expressed and purified from *E. coli* were extracted and analyzed by LC–MS/MS. Samples were spiked with internal standards prior to analysis. Lipids were separated on a C18 column and quantified on a Sciex QTrap5500 LC/MS System operating in multiple reaction monitoring (MRM) mode. Peak areas were normalized to internal standards, and lipid abundances are reported as μg/mg protein. PE 34:1 and PE 36:2 are 2 species of phosphatidylethanolamine; PG 32:1 and PG 34:1 are 2 species of phosphatidylglycerol.

We used a liposome pelleting assay as a second way to demonstrate PIP binding. For this purpose, we expressed and purified from *E. coli*, 6His-PX, and liposomes containing as much as 40% PIP3 (3,4,5). As shown in Supplementary Fig. 5, we could detect binding, but just above background. We next tested the idea that the minimal binding was due to the PX domain already having lipids bound to it, having picked them up during expression and purification from *E. coli*. As shown in Fig. 3, mass spectrometry confirmed our speculation: 6His-PX was bound to several lipids including phosphatidylethanolamine (PE 34:1, and PE 36:2), and phosphatidylglycerol (PG 32:1, and PG 34:1). At the least, these results demonstrate that the PX domain of RRC-1 binds to phospholipids.

**Fig. 4.**
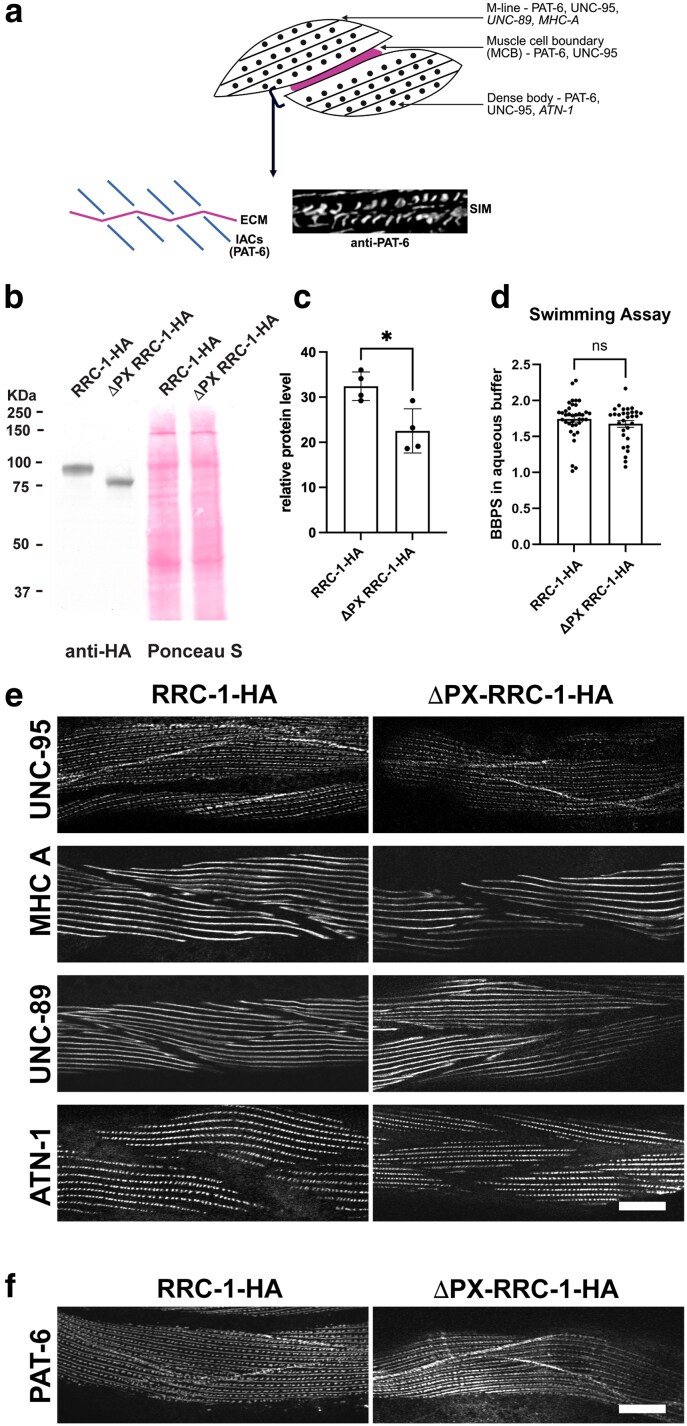
In-frame deletion of the PX domain of RRC-1 results in a normal muscle phenotype. a) Depiction of structures containing IACs in body wall muscle cells. On top is an approximate drawing of 2 adjacent body wall muscle cells, indicating the rows of dense bodies, M-lines, and a muscle cell boundary (MCB). The IAC proteins PAT-6 (parvin) and UNC-95 are present at all 3 structures, whereas UNC-89 (obscurin) and MHC-A (myosin heavy chain) (in italics) are found only at M-lines, and ATN-1 (actinin) (italics) is found only at dense bodies. Below on the left is a drawing of an MCB, which consists of a thin layer of ECM (exaggerated in the top drawing), and within adjacent muscle cells, integrin and integrin-associated proteins like PAT-6 are organized in a “zipper-like” pattern. Below on the right is a super-resolution (SIM) image of an MCB immunostained with antibodies to PAT-6, a type of image that is interpreted by the drawing on the left. b) Western blot demonstrating expression of full-length RRC-1 with an HA tag (RRC-1-HA), and RRC-1 with an in-frame deletion of the PX domain (ΔPX RRC-1-HA), both strains generated by CRISPR/Cas9. c) By quantitative Western blot, ΔPX-RRC-1-HA has a slightly lower level of expression than does full-length RRC-1-HA. *P = 0.0187. d) In a swimming assay, ΔPX RRC-1-HA worms swim as well as RRC-1-HA worms, which swim as well as wild-type worms (Moody et al. 2024). Body bends per second (BBPS) were quantified for individual animals (dots) of each strain. e) Immunostaining with antibodies to various sarcomeric proteins shows that ΔPX-RRC-1-HA worms have normal sarcomere organization, similar to RRC-1-HA worms. Scale bar, 10 µm. f) The IAC component PAT-6 (α-parvin) localizes normally to MCBs in both ΔPX-RRC-1-HA and RRC-1-HA worms. e) and f) display representative images from a minimum set of 10 randomly selected worms for each antibody staining. Scale bar, 10 µm.

To determine the function of RRC-1's PX domain, we used CRISPR to delete in-frame the coding sequence for the PX domain. This was done in our previously reported (Moody et al. 2024) CRISPR strain that expresses RRC-1 with an HA tag at its C-terminus, *rrc-1(syb4499)*. As shown in Fig. 4b, we observe expression of ΔPX RRC-1-HA, and it is of expected size. Also, the level of ΔPX RRC-1-HA is only slightly reduced compared to the level of the full-length RRC-1-HA (Fig. 4c). Worms expressing ΔPX RRC-1-HA show the same locomotion as worms expressing full-length RRC-1-HA (Fig. 4d), which itself did not show a different motility than wild type (Moody et al. 2024). This contrasts with intragenic deletions of *rrc-1* that display reduced swimming and crawling speeds (Moody et al. 2024). Similarly, in contrast to intragenic deletions of *rrc-1*, which result in sarcomere disorganization and lack of accumulation of IAC components at the MCB (Moody et al. 2024), ΔPX-RRC-1-HA displays normal sarcomere organization and IAC localization at the MCB (Fig. 4e and 4f).

**Fig. 5.**
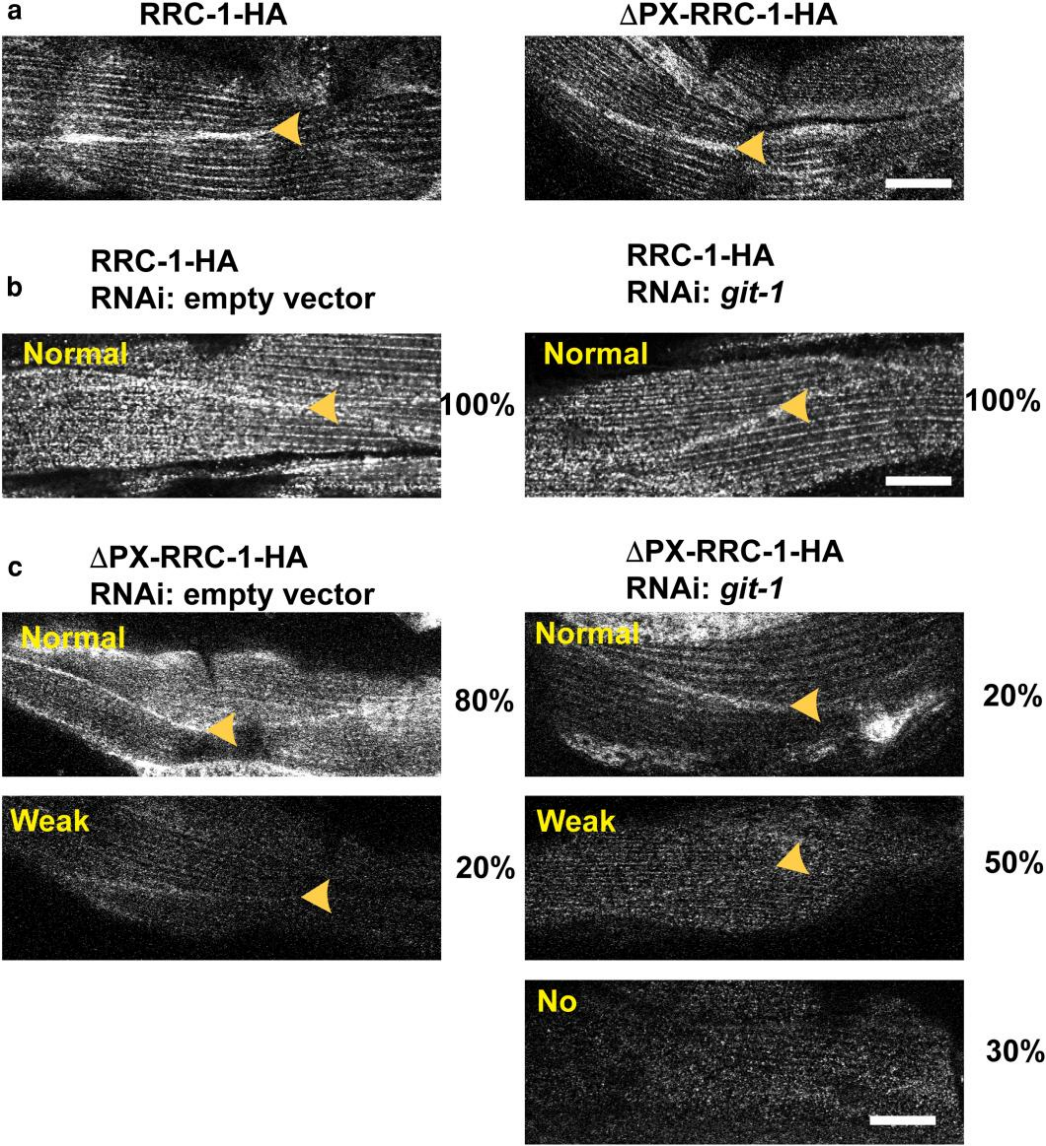
Optimal localization of RRC-1 to the MCB requires both the RRC-1 PX domain and the scaffold protein GIT-1. a) Nematodes expressing either full-length RRC-1-HA or ΔPX-RRC-1-HA show normal localization of RRC-1-HA and ΔPX-RRC-1-HA to the MCB, using antibodies to HA. b) RNAi-knockdown of the PIX scaffold protein GIT-1 does not affect the localization of full-length RRC-1-HA to the MCB. Under each condition, 100% of 10 randomly selected worms examined yielded the same result. c) ΔPX-RRC-1-HA subjected to the RNAi empty vector shows normal localization of ΔPX-RRC-1-HA to the MCB at a frequency of 80%, and weak localization at 20% of the animals. However, when ΔPX-RRC-1-HA animals are subjected to *git-1*(RNAi), ΔPX-RRC-1-HA localizes normally at a frequency of 20%, weakly at a frequency of 50% and not at all at a frequency of 30%. Percentages are based on examining 20 animals. Arrowheads point to MCBs. Scale bars, 10 µm.

ΔPX RRC-1-HA localizes to MCBs and “fuzzily” to dense bodies and M-lines like full-length RRC-1-HA (Fig. 5a). We have reported (Moody et al. 2024) that RRC-1 is part of the PIX complex, which includes the scaffold protein GIT-1. Furthermore, we showed that by RNAi knockdown of *git-1*, GIT-1 is not required for the localization of RRC-1 (also shown in Fig. 5b) but is required for the stability of RRC-1. One caveat is that RNAi usually does not achieve the null state. We did not examine the localization of RRC-1-HA in the deletion allele of *git-1*, *ok1848*, because the 2 genes are less than 1 cM apart, and was more convenient to use RNAi. It is possible that in the null state for *git-1*, RRC-1-HA will be mis-localized. Nevertheless, because the PX domain is not required for RRC-1's localization to the MCB (Fig. 5a), and GIT-1 is apparently also not required for RRC-1's localization to the MCB, we wondered if the PX domain and GIT-1 were redundant for MCB localization. To test this idea, we conducted RNAi for *git-1* in the strain that expresses ΔPX-RRC-1-HA (Fig. 5c). ΔPX-RRC-1-HA with the RNAi empty vector control showed normal localization to the MCB at a frequency of 80%, and weak localization at 20%. In contrast, ΔPX-RRC-1-HA with *git-1(RNAi)* showed normal localization to the MCB at only 20% frequency, weak localization at 50% and no localization at 30%. Therefore, consistent localization of RRC-1 to the MCB requires both its PX domain and GIT-1.

**Fig. 6.**
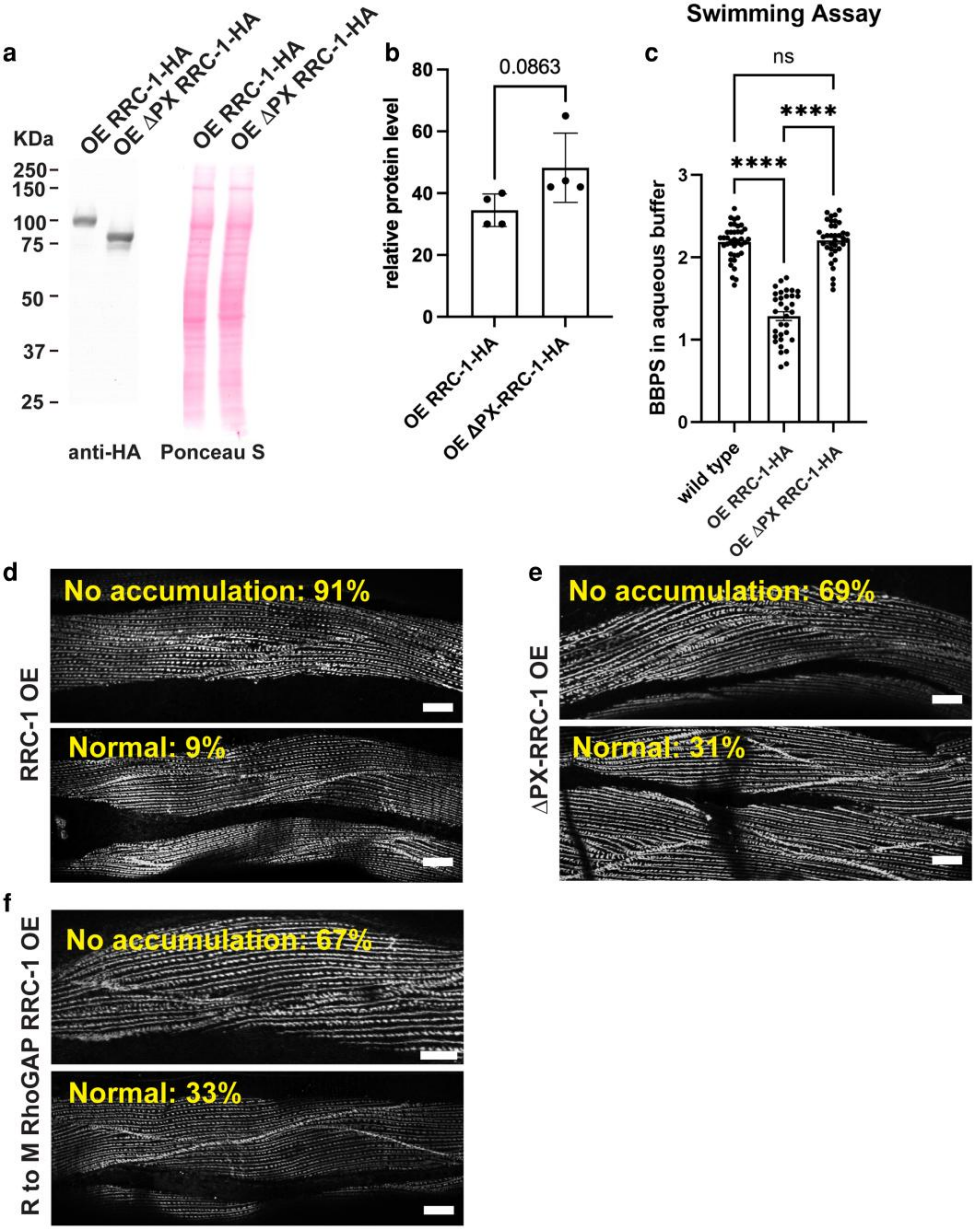
Overexpression experiments indicate that both the PX domain and the RhoGAP domain each contribute to the function of RRC-1 at the MCB. a) Western blot demonstrating overexpression of HA-tagged full-length RRC-1 (OE RRC-1-HA) or RRC-1 with an in-frame deletion of its PX domain (OE ΔPX RRC-1-HA) in body wall muscle from genome-integrated transgenic arrays. b) Western blots reacted with anti-HA indicate no significant difference in the levels of expression from these 2 integrated transgenes (*P* = 0.0863). c) Swimming assays show that overexpression of full-length RRC-1 but not RRC-1 with internal deletion of its PX domain results in reduced whole animal locomotion. The indicated transgenes were integrated into the genome by UV irradiation and outcrossed 3X to wild type prior to conducting the swimming assays. Statistical significance was assessed by Dunnett's T3 multiple comparisons test. ****P < 0.0001; ns: not significant. d) Overexpression of full-length RRC-1 results in no accumulation of the IAC component PAT-6 at the MCB in 91% of the animals (*N* = 11). e) Overexpression of ΔPX-RRC-1 results in no accumulation of PAT-6 at the MCB in 69% of the animals (*N* = 16). f) Overexpression of full-length RRC-1 with a missense mutation inactivating the RhoGAP domain results in no accumulation of PAT-6 at the MCB in 67% of the animals (*N* = 15). Results shown in d), e), and f) were obtained from extrachromosomal arrays expressing the indicated proteins. Arrowheads denote portions of MCBs. Scale bar, 10 µm.

We have reported that both deficiency and overexpression of PIX-1 result in a lack of accumulation of IAC components to the MCB and reduced whole animal locomotion (Moody et al. 2020). We next asked whether overexpression of RRC-1 would have a similar effect and if it depends on the presence of its PX domain. To address this question, we created integrated arrays that overexpress in body wall muscle, using the *myo-3* promoter, full-length RRC-1-HA or ΔPX RRC-1-HA in a wild-type background. As shown in Fig. 6a, a Western blot shows appropriately sized proteins are produced from the respective transgenic lines. In addition, the levels of expression are comparable (Fig. 6b). However, by a swimming locomotion assay, overexpression of full-length but not ΔPX-RRC-1 results in reduced swimming (Fig. 6c). Consistent with this result, overexpression of full-length RRC-1-HA results in reduced accumulation of 2 IAC components, PAT-6 (α-parvin) and UNC-95 (Supplementary Fig. 6). However, overexpression of ΔPX RRC-1-HA shows normal accumulation of PAT-6 and UNC-95 to the MCB in some animals (Supplementary Fig. 6). To quantify this effect, we preferred to use the extrachromosomal array rather than the integrated array to avoid any phenotypic effects from the genomic insertion of the array. We prepared 3 extrachromosomal lines; full-length RRC-1, ΔPX-RRC-1, and RRC-1 with an RtoM mutation predicted to inactivate the RhoGAP domain (Moody et al. 2024). The later array was reported in Moody et al. (2024) and showed that it failed to rescue a *rrc-1* deletion mutant. The ΔPX-RRC-1 line was used to test for the necessity of the PX domain, and the RtoM mutant was used to test for the necessity of RhoGAP activity. As shown in Fig. 6d, overexpression in a wild-type background of full-length RRC-1 eliminated accumulation of PAT-6 at the MCB in 91% of the animals. In contrast, overexpression of RRC-1 lacking its PX domain (Fig. 6e), or overexpression of RRC-1 with an inactivated RhoGAP domain (Fig. 6f), resulted in no accumulation of PAT-6 at the MCB in 69 and 67% of the animals, respectively. These results indicate that both the PX domain and the RhoGAP activity of RRC-1 are each required equally for RRC-1 function at the MCB.

## Discussion

We have reported that RRC-1 is required for IAC assembly, and that the RRC-1 protein is likely a component of at least one type of IAC because it localizes to muscle cell boundaries (MCBs) and exists in a complex with another IAC complex protein, PIX-1 (Moody et al. 2024). In the current study, our prediction of a PX domain in RRC-1 (Fig. 1) and in vitro binding experiments (Fig. 2, Supplementary Fig. 4) indicate that this PX domain binds multiple phosphoinositides (PIPs), consistent with classification as a class IV PX domain (Chandra et al. 2019). Class IV PX domains contain both canonical and secondary phosphoinositide-binding sites, allowing binding of PtdIns3P at the canonical site and PIP2/PIP3/PS at the secondary site (Chandra et al. 2019). Although the exact phosphoinositide-binding interface for the PX domain of RRC-1 remains unknown, our bioinformatic analysis identifies an extended basic surface patch consistent with the secondary PIP-binding interface and a conserved surface patch containing Arg38 that may represent the canonical PtdIns3P-binding site described in other class IV PX domains (Fig. 1) (Ellson et al. 2002; Karathanassis et al. 2002; Chandra and Collins 2018; Chandra et al. 2019). Nevertheless, the precise phosphoinositide-binding interface remains unknown, and we therefore did not attempt mutational studies to abolish PIP binding. Previous mutagenesis studies of PX domains showed that single mutations usually have little effect and that multiple mutations are often required, with binding sometimes persisting even after extensive mutagenesis (Chandra et al. 2019). We therefore expect that abolishing binding in the RRC-1 PX domain would require extensive multi-site mutagenesis, which risks disrupting the domain fold and makes binding experiments difficult to interpret. Without a crystal structure and relying only on an AlphaFold model, we think that such a strategy is unlikely to be successful at present. In addition to the PIP strip assay, we sought an additional way to demonstrate PIP binding using a liposome pelleting assay with 6His-PX and liposomes containing as much as 40% PIP3. We could detect binding, but just above background (Supplementary Fig. 5). We speculated that the minimal binding was due to the PX domain already having lipids bound to it, having picked them up during expression in *E. coli*. Mass spectrometry confirmed our prediction: 6His-PX was bound to several lipids, including phosphatidylethanolamine (34:1 and 36:2), and phosphatidylglycerol (32:1, and 34:1) (Fig. 3). Our combined results demonstrate that the PX domain of RRC-1 binds to phospholipids.

These results are further evidence for the importance of PIPs and their metabolism in IAC assembly. Several IAC components in mammals, all of which have nematode orthologs, bind to PIPs, and this is required for their membrane localization and assembly into IACs. For example, kindlin binds to PIP2 and PIP3 through its PH domain, and at least for kindlin 3, with higher affinity to PIP3 (Liu et al. 2011; Ni et al. 2017). Talin binds to PIP2 via its FERM domain (Chinthalapudi et al. 2018). PIP2 is thought to recruit kindlin and talin to the cell membrane and promote localized phase separation of newly forming IACs (Hsu et al. 2023). Vinculin binds to PIP2 via basic amino acids at its C-terminal region (Thompson et al. 2017). The known final component of the PIX pathway, PAK, via a positively charged region binds to PIP2 of the cell membrane and enhances the kinase activity of already activated GTP-Rac or GTP-Cdc42-bound PAK (Strochlic et al. 2010). Recently, we have shown that IPMK-1 (inositol phosphate multikinase), which converts PIP2 to PIP3, is required for normal IAC assembly in *C. elegans* muscle (Sagadiev et al. 2026).

Our results also indicate that the PX domain of RRC-1, although not essential, is still important for RRC-1 function. Analysis of a worm expressing RRC-1 with an in-frame deletion of PX, shows a level of RRC-1 protein that is not appreciably reduced, and displays normal locomotion, normal sarcomere organization and normal localization of IAC components (Fig. 4). However, RRC-1's localization to the muscle cell boundary, and likely membrane localization, requires 2 factors—RRC-1's PX domain and the PIX complex scaffold protein, GIT-1 (Fig. 5). We have reported that RRC-1 is a member of the PIX complex of proteins, although we have not demonstrated that RRC-1 binds directly to GIT-1 (Moody et al. 2024). In a wild-type background, ΔPX-RRC-1-HA localizes to the MCB and probably the bases of M-lines and dense bodies, just like full-length RRC-1-HA (Fig. 5a). Although with low penetrance (∼20%) we observed reduced accumulation of ΔPX-RRC-1-HA in wild-type animals, this defect was enhanced to 80% in *git-1* RNAi knockdown animals. This dual requirement for proper RRC-1 localization likely is via membrane association—via the PX domain of RRC-1 and via the ARFGAP domain of GIT-1, since ARFGAP domains may bind to PIP3 (Czech 2000). Overexpression of full-length RRC-1 results in reduced accumulation of IAC components at the MCB in nearly all (91%) of the animals (Fig. 6d). The fraction of animals which have reduced accumulation of IAC components at the MCB is reduced to 69% if overexpressed RRC-1 is missing its PX domain (Fig. 6e). Similarly, the fraction of animals having reduced accumulation of IAC components at the MCB is reduced to 67% if overexpressed RRC-1 has an inactivated RhoGAP domain (Fig. 6f). These results suggest that both the PX domain and the RhoGAP domain are equally but partially important for RRC-1's function at the MCB.

There are 2 possibilities for the phenotypes resulting from overexpression of these 3 forms of RRC-1: (i) For the full-length RRC-1 or RRC-1 with an inactive RhoGAP domain, there is a titration or mass action effect—too much of the PIP-binding PX domain could compete out the binding to PIPs and membrane localization of key IAC components like kindlin, talin, and vinculin. (ii) For the full-length RRC-1 or RRC-1 missing its PX domain, there is too much RacGAP activity—the extra RRC-1 localizes to the muscle cell membrane, and there is consequently increased RacGAP activity locally, which results in reduced levels of active GTP-bound Rac, similar to loss of function of the RacGEF PIX-1 (Moody et al. 2020).

## Supplementary Material

jkag143_Supplementary_Data

## Data Availability

Worm and bacterial strains, and plasmids are available upon request. The authors affirm that all data necessary for confirming the conclusions of the article are present within the article, figures, and Supplementary Material. Supplemental material available at G3 online.
