# Supplementary material for: The lipid-binding PX domain of RRC-1 (ARHGAP32/33) is required for optimal assembly and function of integrin adhesion complexes at the muscle cell boundary in C. elegans
Source: G3 (Bethesda). 2026 Jun 1;16(8):jkag143. doi: 10.1093/g3journal/jkag143 (PMC13439933; doi:10.1093/g3journal/jkag143)
Supplement: Supplementary file 1 [file jkag143_supplementary_data.zip › Supplemental_Material_Legends_G3-2026-406812.docx]

**Supplemental Material Legends**

**The Lipid-binding PX Domain of RRC-1 (ARHGAP32/33) is Required for Optimal Assembly and Function of Integrin Adhesion Complexes at the Muscle Cell Boundary in *C. elegans***

Sara Sagadiev^1^, Isabel Martin^2^, Nahum Arefeayne^1^, Yuchu Wang^1^, Robert Hudson, Jr.^1^, Olga Mayans^2^, Hiroshi Qadota^1^*, Guy M. Benian^1^*

^1^ Department of Pathology, Emory University, Atlanta, Georgia, 30322 USA

^2^ Department of Biology, University of Konstanz, 78457 Konstanz, Germany

* co-corresponding authors, [hkadota@emory.edu](mailto:hkadota@emory.edu), [pathgb@emory.edu](mailto:pathgb@emory.edu)

**Supplementary Figure 1. Generation of strain PHX10067 [rrc-1(syb4499 syb10067)] which expresses RRC-1 with a C-terminal HA tag and has an in-frame deletion of the coding sequence for the PX domain.** This strain was created by SunyBiotech, beginning with PHX4499 which expresses full-length RRC-1 with a C-terminal HA tag (Moody et al. 2020).

**Supplementary Figure 2. AlphaFold model of the RRC-1 PX domain.** 3D-fold of the RRC-1 PX domain (residue 41-146) predicted by Alphafold3. The top model (model B) is shown as a colored ribbon, where color corresponds to the per-residue pLDDT confidence score. The subsequent best 5 models are depicted as transparent ribbons. A pLDDT score above 90 (dark blue) indicates high confidence in the predicted model.

**Supplementary Figure 3. Structure-based multiple sequence alignment of the RRC-1 PX domain and its ten closest structural homologues reveals conserved residues in low sequence similarity structures.** The proteins used for the structural alignment are listed with their respective PDB code. Residue boundaries for each sequence are given in brackets. Nomenclature: CIS = cytokine-independent survival; PI = phosphatidylinositol; H = Homo sapiens; M = Mus musculus; T = Thermochaetoides thermophila). Secondary structure elements are marked. Conservation score is given as: * = all residues in that column are identical; : = conserved substitutions; . = semi-conserved substitution. Residues potentially forming the secondary PIP2/PIP3-binding site are highlighted in pink, while the single arginine corresponding to the canonical PtdIns3P-binding site is highlighted in yellow, as described by Chandra et al. (2019).

**Supplementary Figure 4. Protein-lipid overlay assay demonstrates that RRC-1 (1-150) binds to phosphoinositides.** Results of incubating GST-RRC-1(1-150) and GST with a PIP Thin Strip (ThermoFisher), and detection using antibodies to GST. Identities of spotted lipids are shown to the left. An independently generated strip was used compared to that shown in Figure 2. No binding was found with GST alone (data not shown).

**Supplementary Figure 5. Liposome pelleting assay.** Liposome pelleting assay of 6His–RRC-1 (36–150 aa). a) Coomassie gel showing co-pelleting of 6His–RRC-1 (36–150 aa) with multilamellar vesicles (MLV) containing different ratios of 1-palmitoyl-2-oleoyl-glycero-3-phosphocholine (PC) and 1,2-dioleoyl-sn-glycero-3-phosphoethanolamine (PE) and with or without PI(3,4,5)P3 (PIP3). S: supernatant and P: pellet. Two negative controls with just 6His–RRC-1 (36–150 aa) but no MLVs (-MLVs) were included. b) Quantitation of the ratio of the protein in the pellet versus supernatant.

**Supplementary Figure 6.** Overexpression from integrated transgenic arrays of full-length RRC-1 but not RRC-1 with internal deletion of its PX domain results in decreased accumulation at the MCB of two IAC proteins, PAT-6 and UNC-95. Shown are images of several body wall muscle cells immunostained with antibodies to either PAT-6 or UNC-95. Arrowheads denote portions of MCBs. Scale bar, 10 μm.

**Supplementary Table 1. Top ten structurally similar PDB entries to the predicted 3D-model of PX-RRC-1 domain as identified by DALI server.** The first column provides protein names (CIS = cytokine-independent survival; PI = phosphatidylinositol). The second column indicates the species of origin for each protein (H = Homo sapiens, M = Mus musculus, and T = Thermochaetoides thermophile). The third column lists the corresponding PDB codes of the crystal structures. The fourth column presents the DALI Z-scores, indicating the quality of the structural matches. The fifth column shows the mean root-mean-square deviation (RMSD) of the structural alignments. The sixth column reports the length of the aligned region, and the seventh column gives the total sequence length of each protein. The final column indicates the sequence identity (%) from pairwise alignments as output by DALI.
