# Supplementary material for: The lipid-binding PX domain of RRC-1 (ARHGAP32/33) is required for optimal assembly and function of integrin adhesion complexes at the muscle cell boundary in C. elegans
Source: G3 (Bethesda). 2026 Jun 1;16(8):jkag143. doi: 10.1093/g3journal/jkag143 (PMC13439933; doi:10.1093/g3journal/jkag143)
Supplement: Supplementary file 1 [file jkag143_supplementary_data.zip › Supplementary_Figure_1_G3-2026-406812.pdf]

Supplementary Figure 1. Generation of strain PHX10067 [rrc-1(syb4499 syb10067)] which expresses RRC-1 with a C-terminal HA tag and has an in-frame deletion of the coding sequence for the PX domain. This strain was created by SunyBiotech, beginning with PHX4499 which expresses full-length RRC-1 with a C-terminal HA tag (Moody et al. 2020).

sgRNA target site:

BG43-Sg1:CCATTATTCGTCTGTTGAATTGG
BG43-Sg2:CATTATTCGTCTGTTGAATTGGG
BG43-Sg3:aaaacacagATTGACTCTCGTGG
BG43-Sg4:acacagATTGACTCTCGTGGCGG

Repair template (oligonucleotide):

BG43-OLIGO:
gTCTTCGCATTCAACGACTAGTCGACTGTCAACACTTCCATTATTCGTCT-
GACTCTCGTGGCGGACATTTTGAACCAGCTGAAGAAACTTCGATCAACGT

-: deletion site

PCR and sequencing primers:

BG43-KO-S:AAGGCGTGAGACTTATGATC
BG43-KO-A:TTTTAAATCAGAATGGGCTC

In the sequences below, the 10 nucleotides before and after the deletion site are underlined to make it clear which sequence was deleted.

DNA sequence of syb10067(-1401bp)
aaggcgtgagacttatgatctcattgggttaaaccagtcgcgggttcgaatcccgcccggggcaatatccttttt
ttactattaaatcaagttttaccaaagaataaccattaaaagaaaaataattcgaactgtacattaaataattgt
aactacttcagcccttttttaaccaagtttaggccactatagaaacattttcagtgcaaagttttcagcgcatccc
caactttaatttattttacccaactttagccagtttttcactaaatgcattcctaaagctgataaggttggtaga
tatgttgcggcgttctttcaagaaaatatctttacatttcttcaccacaaacattttaattggaatattaatgtt
ttagtatcaactttgtgagttgaaaaatttttgaaagaagcaaattttgctagctggaaataattcatatactac
ttcactgtttatttcgttgaatatcaacaatttattgtgtttcagTCTTCGCATTCAACGACTAGTCGACTGTCA
ACACTTCCATTATTCGTCT-
GACTCTCGTGGCGGACATTTTGAACCAGCTGAAGAAACTTCGATCAACGTGCCAGCCATCGCAGCTGCAGTAGTC
ACAAAGGATTTTGAGCCGACCGAAAGCTCACAGCTGCGACTGCGGgtatctttcaaatttggcccggcgactaca
agtgtaaacaaaaacattgcagGTCGGTGATATCGTCTCAATTACGGAAATGTCAACAGCAAGTCCCAGCGAGCA

AACATTTTGGAAAGCAAAACTGACTATATCCAACCAGAAGATTGTAGATCCGCAAAATGCACGGCTAGGTTTTGA
Agtaagtctatttaattattattaattggaaatgttgtatcaacgtgcctttggctcttctgaacttaaaattgc
aaaagttttggttaatgctaagtaattatacgaagatttttgttcaacatcttttctaagagcccattctgattt
aaaa

DNA sequence of syb4499

aaggcgtgagacttatgatctcattgggttaaaccagtcgcgggttcgaatcccgcccggggcaatatccttttt
ttactattaaatcaagttttaccaaagaataaccattaaaagaaaaataattcgaactgtacattaaataattgt
aactacttcagcccttttttaaccaagtttaggccactatagaaacattttcagtgcaaagttttcagcgcatccc
caactttaatttattttacccaactttagccagtttttcactaaatgcattcctaaagctgataaggttggtaga
tatgttgcggcgttctttcaagaaaatatctttacatttcttcaccacaaacattttaattggaatattaatgtt
ttagtatcaactttgtgagttgaaaaatttttgaaagaagcaaattttgctagctggaaataattcatatactac
ttcactgtttatttcgttgaatatcaacaatttattgtgtttcagTCTTCGCATTCAACGACTAGTCGACTGTCA
ACACTTCCATTATTCGTCTGTTGAATTGGGTCCCGTCCGTGTTGCCATCATCGCAATAAACGCTGACGAGAATGC
AACAGAGCGGATAAAAATGCGTGTGGAAAGTGAATCAAATTCATGgtgagttggtcattccttctgtttccaatt
tgtcttcaaaaattcgtgctccgtggtgtttctctcggtttttctttcaatgttctgccacacagaaatcaaaca
aaaaagccacgagtgaagtctcatgggagatgagaataaaggctgaataaagttagtacgaggaagaacaaaaaa
gaaaaaagaaagaaaaacggaaacttgaaaggttttccatcattgattcattcagGCTTGTCGAACGTTCTCGGG
AGGATTGGGCAGTCTTCGACAGACAGCTGCATCGTTGTGTGTTCGAACGAAGGCATTCACGACTTGACGAGCTGT
TTCCTCTTATTCATCTGGAAACGGCAAAGTTCGAGgtgacggcttcttttcaaaatcactagtcatcacatttga
tgtttacagGAAGTTCTTGTCAAGTATACGGAGCGATTATCGGAACTCACTGGCAGCATTATAACGTGCTATCCT
GTTCTGAAATTTTTAGAAgtaagcgaaattgtttttaactccaaacttcaatttaatttaaatttaaaactggct
atggtttagacttttttacagtatctcttcaaattagaaaagttaaattaaaaaagattcgaataaaccaaatgaa
cccaagacgataaatatttcatttcaagtattgcaaacatttcagataggaaaatttgttttcctttctgacaga
atagtaagtctgtatttcaaagagttattcaattatttgaacctttctcacaaaattggtgagttctagtacaac
cttgaaaaaatatcttgttcttcttatgatttttgacaccactcgctcttaactgcagtgcatatttcatttctgt
gtttgtttttattgcaaaacatatgtacaatgtaatgcacacctacattatgcattagtatcaggacttcgtcaa
acaagtttttttttcattctttttgcatttgtttcagtcagttctgaaactgttctctcaaactatcacttaaca
aaaaattacgatcttctaaatgtagcccaaaacgttttcagttgaaattgaaaacgttcatgcgttatttttcaa
acctgcggaataatcgttttttataaataagtatttacacgcttttttattacatagacaaacattccagaatgaac
tactccaattctttctaattcactaaactttctgagttatttcgattgatatagctattttatttaataggaatt
atagttgtactaaatttcgacaatcctttcattatttctcttacgcgcataatctctgaaaacacagATTGACTC
TCGTGGCGGACATTTTGAACCAGCTGAAGAAACTTCGATCAACGTGCCAGCCATCGCAGCTGCAGTAGTCACAAA
GGATTTTGAGCCGACCGAAAGCTCACAGCTGCGACTGCGGgtatctttcaaatttggcccggcgactacaagtgt
aaacaaaaaacattgcagGTCGGTGATATCGTCTCAATTACGGAAATGTCAACAGCAAGTCCCAGCGAGCAAACAT
TTTGGAAAGCAAAACTGACTATATCCAACCAGAAGATTGTAGATCCGCAAAATGCACGGCTAGGTTTTGAAgtaa
gtctatttaattattattaattggaaatgttgtatcaacgtgcctttggctcttctgaacttaaaattgcaaaag
ttttggttaatgctaagtaattatacgaagatttttgttcaacatcttttctaagagcccattctgatttaaaa
